# Economic crisis and health inequalities: evidence from the European Union

**DOI:** 10.1186/s12939-016-0425-6

**Published:** 2016-09-01

**Authors:** Laia Maynou, Marc Saez

**Affiliations:** 1 Center for Research in Health and Economics (CRES), Universitat Pompeu Fabra, C/Ramon Trias Fargas, 25-27, Mercè Rodoreda Building, 08005 Barcelona, Spain; 2 Research Group on Statistics, Econometrics and Health (GRECS), Universitat de Girona, Campus de Montilivi, 17071 Girona, Spain; 3 CIBER of Epidemiology and Public Health (CIBERESP), Madrid, Spain

**Keywords:** Economic crisis, Health inequalities, Dynamic panel model, Random-effects, σ-convergence, I14, I15, C33, C11

## Abstract

**Background:**

The recent economic crisis has been a major shock not only to the economic sector, but also to the rest of society. Our main objective in this paper is to show the impact of the economic crisis on convergence, i.e. the reduction or equalising of disparities, among the EU-27 countries in terms of health. The aim is to observe whether the economic crisis (from 2008 onwards) has in fact had an effect on health inequalities within the EU.

**Methods:**

We estimate convergence by specifying a dynamic panel model with random-effects (time, regions and countries). We are particularly interested in σ-convergence. As dependent variables, we use life expectancy, total mortality and (cause-specific) mortality in the regions of the EU-27 countries over the period 1995–2011.

**Results:**

The results of the analysis show that, in terms of health, there has been a catching-up process among the EU regions. However, we find no reduction, on average, in dispersion levels as the σ-convergence shows. The main finding of this paper has been the sharp increase in disparities in 2010 for all health outcomes (albeit less abrupt for cancer mortality).

**Conclusion:**

This increase in disparities in 2010 coincides with the austerity measures implemented in the EU countries. Our main conclusion is that these austerity measures have had an impact on socioeconomic inequalities.

## Background

The recent economic crisis has been a major shock not only to the economic sector, but also to the rest of society. Since 2008, a weakening in commodity demand has lead to economic recession, which in turn has resulted in increased unemployment and reduced economic growth. European governments have implemented various measures, mainly focused on cutting public spending, the privatization of public services and market deregulation, to stabilise the economy and overcome the crisis [1]. The impact of the crisis on Europe and, in particular, the consequences of these very policies, has been the incentive behind this study into the potential impact they have had on socioeconomic inequalities [2–5].

Although Atkinson and Morelli [6] found evidence of financial crises increasing inequalities, they were unable to determine a clear pattern as each crisis has its own characteristics. However, there has been increasing interest in going into more detail and analysing the impact on health inequalities only. The literature shows evidence of an increase in health inequalities during crisis periods, both previous [7, 8] and current [9–12]. These health inequalities have been seen in different health variables: mortality, mental health, self-perceived health, excessive alcohol consumption, health-related quality of life, long-standing illness, and disability (for review see [13]). However, some studies of previous crises, mainly focused on Nordic countries, do not confirm this evidence [14–19].

In this paper, we attempt to assess the effect of the economic crisis on health inequalities by focusing on the analysis of σ-convergence. According to this hypothesis, σ-convergence exists if dispersion and inequalities between countries are reduced over time [20]. In Maynou et al. [21] we made use of this convergence hypothesis to approximate health inequalities. In particular, we analysed convergence using life expectancy and (cause-specific) mortality in the European Union (EU-27) regions from 1995 to 2009. We show that, rather than converging, health inequalities increased during the studied period. Moreover, out of the research presented at Maynou et al. [21], we move a bit further and we talk about socioeconomic inequalities in health, instead of only health inequalities, due to the variables that are used in this analysis. In order to perform the study, even if we are using health indicators, these factors can be related to socioeconomic elements, as defined in the literature [22–24] they are good proxies. This fact allows as moving the concept to socioeconomic inequalities in health.

Our objective in this paper is to contribute to the above literature and show the impact of the economic crisis on socioeconomic inequalities in health. Here, making use of the methodology developed in Maynou et al. [21], the impact is analysed through the σ-convergence hypothesis (i.e. the reduction or equalising of disparities) among the EU-27 in the period 1995–2011. This paper differs from the previous one, by assessing socioeconomic inequalities in health in a particular time period (crisis) with the aim of relating this effect with the previous literature.

The paper is organised as follows. We define the methodology in Section Methods. The results of the model are explained and discussed in Section Results. Finally, we conclude in Section Discussion.

## Methods

### Data setting

We use data from 271 regions of the 27 EU member countries (all members except Croatia) from 1995 to 2011. In particular, the countries included in the study are: Austria, Belgium, Bulgaria, Republic of Cyprus, Czech Republic, Denmark, Estonia, Finland, France, Germany, Greece, Hungary, Ireland, Italy, Latvia, Lithuania, Luxembourg, Malta, Netherlands, Poland, Portugal, Romania, Slovakia, Slovenia, Spain, Sweden and the UK. The years analysed are constraint to data availability. Data are obtained from EUROSTAT [25].

### Econometric model

Although models are specified based on the well-known β-convergence hypothesis [26–29], in the form of the conditional specification of the β-convergence hypothesis, in contrast to more standard studies, we do not specify cross-section, but rather spatio-temporal models, i.e. a dynamic panel model. Furthermore, we are not only interested in the (conditional) β-convergence, but also in the σ-convergence.

In particular, we have specified the following model:

1 $$ \begin{array}{l} \log \left({y}_{ijt}\right)={\alpha}_i+{\beta}_{jt} \log \left({y}_{ijt-1}\right)+{\gamma}_{1jt} \log \left( gdpp{c}_{jt}\right)+{\gamma}_2 \log \left( gdpp{c}_{jt-1}\right)+\\ {}{\gamma}_3 \log \left( gdpp{c}_{jt-2}\right)+{\gamma}_{4jt} \log \left( Gin{i}_{jt}\right)+{\gamma}_{5jt} \log \left( Gin{i}_{jt-1}\right)+{\gamma}_6 \log (empht)+\\ {}{\gamma}_7 \log \left(uni{v}_{ijt}\right)+{\gamma}_8 \log \left(um{y}_{ijt}\right)+{\gamma}_9 \log \left(uf{y}_{ijt}\right)+{\gamma}_{10} \log \left( rand{d}_{jt}\right)+{\gamma}_{11} \log \left({\mathrm{bpg}}_{jt}\right)+\\ {}{\gamma}_{12} \log \left( pub{ \exp}_{jt}\right)+{\gamma}_{13}\left(I>2003\right)+{\gamma}_{14}\left(I>2006\right)+{\gamma}_{15}\left(I>2007\right)+{S}_i+{\tau}_t+{u}_{ijt}\end{array} $$

2 $$ \begin{array}{l} \log \left( Gin{i}_{jt}\right)={\delta}_{0j}+{\delta}_1 \log \left({y}_{jt-1}\right)+{\delta}_2 \log \left({y}_{jt-2}\right)+{\delta}_3 \log \left({y}_{jt-3}\right)+{\delta}_4 \log \left( gdpp{c}_{jt-1}\right)+{\delta}_5 \log \left( gdpp{c}_{jt-2}\right)+\\ {}{\delta}_6 \log \left( gdpp{c}_{jt-3}\right)+{\delta}_7 rat{e}_{jt-1}+{\delta}_8 \log \left( Gin{i}_{jt-1}\right)+{\delta}_9\left(I>2003\right)+{\delta}_{10}\left(I>2006\right)+{\delta}_{11}\left(I>2007\right)+\\ {}{S}_j^{\prime }+{\tau}_j^{\prime }+{v}_{jt}\end{array} $$

Where *y* denotes one of the five dependent variables we chose: life expectancy at birth (in years); mortality for all causes; and cause-specific mortality: ischemic heart disease mortality; cancer mortality; and larynx, trachea, bronchus and lung cancer mortality (cause-specific mortality was standardised as death rate per 100,000 inhabitants, 3-year average). The theoretical explanation behind the use of these variables is the following. First, as in most previous studies on health (in concurrence with the seminal article of Sen et al. [30]), we use life expectancy at birth (in years). However, instead of using only total mortality, we prefer to use here (several) cause-specific mortality. Total mortality is actually a combination of many phenomena that could undermine this variable as an indicator of social ill-being [31]. In particular, we chose those causes of mortality most associated with socioeconomic deprivation in the literature [22–24]: ischaemic heart disease mortality; cancer mortality; and larynx, trachea, bronchus and lung cancer mortality.

The Gini index is one of the main explanatory variables of this model. According to Eurostat [25], it is defined as the relationship of cumulative shares of the population arranged according to the level of equivalized disposable income to the cumulative share of the equivalized total disposable income received by them. More conveniently, it can be defined as twice the covariance between income and income ranks. Note that, because there could be bidirectional causation between health variables (i.e. dependent variables) and income inequality, the Gini index (the main explanatory variable in Eq. (1)), could be an endogenous variable. Even if there exist controversy across authors about this bidirectional causation, evidence (few papers) shows that unhealthy societies can have an important effect on a persistent low economic growth and, maybe, inequality [32, 33]. Moreover, the macroeconomic theory says that the countries with poorer health conditions have more difficulties to reach a sustained economic growth in comparison to other countries with better health [34]. For this reason we specify a model of simultaneous equations.

The subscript *i* denotes region (*i = 1,…,273*); *j* country (*j = 1,…,27*); and *t* year (*t = 1995 1996,…, 2011*); α, β and γ denote unknown parameters; *S* denotes spatial random-effects (see below); and *u* normally distributed disturbance term. Some data is missing for the five dependent variables mainly for the beginning of the period and specifically for some regions in Belgium, Denmark, Italy, Poland, Romania and Slovenia.

Socioeconomic inequalities in health are approached by the Gini index (Gini) (data available only on country level) and the Gross Domestic Product per capita (GDP per capita, (gdppc)) (data available regionally). Note that we assume that the effects, if any, of GDP per capita on socioeconomic inequalities in health, are distributed in time. Hence, we include the current level (*t*) and two lags (*t-*1 and *t-2*) of GDP per capita (gdppcjt-1 and gdppcjt-2). In the equation corresponding to the Gini index (Eq. (2)) we include, additionally, the lag of the growth rate of GDP (rate).

Moreover, we also consider additional variables that may secondarily contribute to socioeconomic inequalities in health. These variables are available on both a regional and country level. The panel that we create with these data is unbalanced. Data was not available for the entire period or for all regions. Further details on the dataset can be found in Maynou et al. [21].

*Regional level:*

**Table:** 

*Empht*: high-tech employment	Employment in technology and knowledge-intensive sectors (thousands of employees), 1999–2011.
*Univ: Percentage of university students*	Ratio of the sum of level 5 and 6 students (tertiary education) over total population from 1999 to 2011. Data is missing for Germany, Greece, Spain and United Kingdom. These countries do not report all data on education to EUROSTAT.
*Umy: Youth male unemployment rate.*	Unemployment rate for young males (15–24 years old) from 1999 to 2011 on average for the regions of the EU. For some regions, some data is missing for some years, mainly for the latter period.
*Ufy: Youth female unemployment rate*	Unemployment rate for young females (15–24 years old) from 1999 to 2011.

*Country level:*

**Table:** 

*RandD*: R&D	Ratio of R&D over the country’s GDP. For some regions, some data is missing for some years, mainly for the first period. Data available from 1995–2011.
*Bpg:* External balance	The ratio of exported goods minus imported goods over the country’s GDP. All data available from 1995 to 2011, except for the first years of the period in Greece.
*Pubexp:* Public expenditure rate	Ratio of goods and services bought by the State over the country’s GDP. All data available from 1995 to 2011.

Finally, we included three dummy variables, taking the value 1 for 2004 onwards (corresponding with the first expansion of the EU in 2003 and so within the study period), for 2007 onwards (corresponding with the second expansion in 2006), and for 2008 onwards (corresponding to the first year of the financial crisis, in 2007).

In order to analyse σ-convergence, we used the coefficient of variation for each health variable. It is important to note, however, that instead of using the coefficient of variation calculated on the original variables, we calculated the fitted values from the model (1-2).^1^

Some of the coefficients have subscripts. In fact, we specify (dynamic) random coefficient panel data models [35] or, in mixed models terminology, we allow (some of the) coefficients to be random-effects [36]. In other words, we have allowed them to be different for the various levels we have considered. Thus, for example, *β,* varies per year,

$$ {\beta}_t=\beta +{\nu}_t $$

and also per country,

$$ {\beta}_{jt}=\beta +{\upsilon}_{jt} $$

With respect to the other explanatory variables, the random-effects are associated with different levels depending on the final model.^2^

When the random-effects vary by country, we assume they are identical and independent Gaussian random variables with constant variance, i.e. *υ*_*jt*_ ~ *N*(0, *σ*_*υ*_^2^). When the random-effects vary by year, we assume a random walk of order 1 (i.e. independent increments) for the Gaussian random-effects vector [37].

$$ \varDelta {\upsilon}_{jt}={\upsilon}_{jt}-{\upsilon}_{jt+1}\kern2em \varDelta {\upsilon}_{jt}\sim N\left(0,{\sigma}_{\upsilon}^2\right) $$

### Spatio-temporal adjustment

We took into account the spatio-temporal extra-variability present in our model (i.e. spatial heterogeneity and spatial and temporal dependence), by introducing some structure into the model. Heterogeneity was captured by using the random-effect associated with the intercept (*α*) (varying on a region, level i in the response variable equation and on a country level j in the Gini equation). Temporal dependency is approximated through the random walk of order 1, and linked to the random-effects associated with the temporal trend (τ in Eqs. (1) and (2)) and also with those parameters varying on a year level, t. Note also, that we allow that this temporal trend to vary per country.

For spatial dependency, we follow the recent work of Lindgren et al. [38], and specify a Matérn structure [39] for the corresponding random-effect (S_i_ or S_j_, in the response variables and in the Gini equation, respectively). In short, we use a representation of the Gaussian Markov Random Field (GMRF) explicitly constructed through stochastic partial differential equations (SPDE) and which has as a solution a Gaussian Field (GF) with a Matérn covariance function [39].

### Inference

We preferred to relax the assumption of strict exogeneity, allowing a weak exogeneity of the lagged dependent variable, that is to say, that current shocks only affect future values of the dependent variable [40]. By doing this, we are able to obtain consistent estimates of the parameters of interest (even with fixed T). It is important to point out that this relaxation involves two requirements, first, a large N: i.e. obtained in our case by considering regional data and second, identically and independently distributed error terms. This can only be achieved by the space-time adjustment explained above, imposing a certain structure on the original disturbance term.

Inferences were performed using a Bayesian framework, following the Integrated Nested Laplace Approximation (INLA) approach [41, 42]. It is important to point out that both equations were estimated simultaneously, avoiding endogeneity.

All analyses are made with the free software R (version 2.15.3) [43], made available through the INLA library [37, 42].

## Results

In Table 1, we provide the descriptive statistics of the variables used in the models. This table collects the mean, the standard deviation, the minimum and the maximum value and the number of observations for each dependent and explanatory variable.

**Table 1:** Descriptive statistics

Variables	Mean	Std. D	Min	Max	Number
Life expectancy	78.14	2.81	67.70	83.30	3286
Total mortality	1003.85	187.40	309.60	2093.40	3578
Ischemic heart disease mortality	109.49	62.56	18.70	414.20	2596
Cancer mortality	180.58	30.47	61.10	477.30	2613
Lung cancer mortality	40.21	10.98	10.20	100.3	2661
GDP per capita in PPS	19474.51	8422.08	3200	81,400	3605
Gini index	29.65	3.64	20	39.20	3339
Employment high tech.	488.36	252.48	9.66	998.04	2457
University students (% population)	22.10	3.72	10.53	37.12	1962
Young male unemployment rate (%)	18	10.09	1.40	60.10	2601
Young female unemployment rate (%)	20.07	13.04	1.90	78.90	2529
R&D (% GDP)	1.24	1.45	0	13.17	2212
External balance (%)	−1.43	6.83	−32.40	27.60	3992
Public expenditure rate (%)	46.52	5.58	31.20	64.90	4065

Source: own construction

The results from estimating the models are shown in Tables 2 and 3. Table 2 shows a negative and significant β for the five models.^3^

**Table 2:** Results of the estimation of the models (fixed effects)

Variables	Life expectancy	Total mortality	Ischemic heart mortality	Cancer mortality	Lung cancer
Intercept	2.2631**^a^ (0.0820)	3.6644** (0.3307)	1.3910** (0.3605)	4.3019** (0.3285)	1.0064** (0.3070)
β Log(dependent_1)	−0.5109** (0.0188)	−0.5258** (0.0468)	−0.2751** (0.0336)	−0.9980** (0.0345)	−0.5075** (0.0410)
Log(gdppc)	0.0000 (0.0001)	0.0041** (0.0008)	0.0025 (0.0036)	0.0008 (0.0018)	0.0005 (0.020)
Log(gdppc_1)	0.000 (0.0001)	−0.0044** (0.0010)	−0.0038 (0.0042)	−0.0039 (0.0022)	−0.0022 (0.0024)
Log(gdp_2)	−0.0001 (0.0001)	0.0003 (0.0007)	−0.0056 (0.0055)	−0.0039 (0.0035)	0.0109** (0.0032)
Gini	−0.1836** (0.0592)	−1.0516 (0.5612)	2.0101 (1.3376)	0.5313 (0.7758)	1.8337** (0.8369)
Gini_1	0.0280 (0.0274)	1.0406** (0.5213)	−1.72 (1.1931)	2.2466** (0.6853)	−0.0302 (0.7540)
Log(empht)	0.001** (0.000)	0.0001 (0.0005)	0.0038** (0.0019)	−0.0027 (0.0014)	−0.0019 (0.0015)
University students (% pop)	−0.0023 (0.0013)	−0.0023 (0.0130)	0.1488** (0.0587)	0.1074** (0.0318)	0.1181** (0.0363)
Young male unemployment (%)	0.0013 (0.0017)	−0.0313 (0.0167)	0.1876** (0.0657)	−0.0219 (0.0363)	0.1075** (0.0402)
Young female unemployment (%)	0.0002 (0.0014)	0.0108 (0.0134)	−0.2707** (0.0524)	−0.1870** (0.0281)	−0.2125** (0.0320)
R&D	0.0078 (0.0091)	−0.0475 (0.0960)	0.4954 (0.3590)	0.0083 (0.1893)	−0.0288 (0.2127)
External balance (%)	0.0237** (0.0038)	0.07 (0.0363)	−0.1082 (0.1749)	−0.1175 (0.1052)	0.0981 (0.1178)
Public expenditure (%)	0.0081** (0.0039)	−0.2011** (0.0427)	−0.3496** (0.1734)	0.1256 (0.0931)	0.1642 (0.1095)
year > 2003	0.0051** (0.0025)	−0.0332** (0.0138)	−0.0107 (0.1082)	−0.0102 (0.0808)	−0.0042 (0.0475)
year > 2006	0.0006 (0.0025)	−0.0132 (0.0139)	−0.0284 (0.1083)	−0.0158 (0.0808)	−0.0121 (0.0476)
year > 2007	0.0011 (0.0025)	0.0286** (0.0139)	−0.0257 (0.1083)	0.0103 (0.0809)	0.0148 (0.0476)
Random-effects					
Region					
Country					
Year					
DIC	−32795.94	−11295.11	−2362.19	−5223.71	−4664.25
Effective number of parameters	3133.15	350.65	227.40	282.85	282.54
CPO	−4.1409	−1.7082	−0.5662	−1.2035	−1.0427

^a^mean (standard deviation). ** Those coefficients where the 95 % credible interval did not contain the zero (statistically significant). Source: own construction

**Table 3:** Results of the estimation of the models (random-effects) ^a^

Variables	Life expectancy	Total mortality	Ischemic heart mortality	Cancer mortality	Lung cancer
*α*_*j*_	Estonia −0.066 (0.025) ^b^	Ireland −0.454 (0.102)	Czech Republic 0.3096 (0.087)	Bulgaria 0.1765 (0.0816)	Czech Republic 0.2767 (0.0812)
	Greece 0.045 (0.014)	Poland 1.213 (0.089)	France −0.1286 (0.0493)	Czech Republic 0.5614 (0.0909)	Germany −0.1319 (0.053)
	Hungary −0.031 (0.015)		Netherlands −0.1132 (0.0479)	Hungary 0.261 (0.1011)	Hungary 0.1596 (0.082)
	Italy 0.027 (0.012)				
	Poland −0.026 (0.011)				
	Portugal −0.045 (0.015)				
	Spain 0.032 (0.012)				
	Sweden 0.038 (0.017)				
*β*_*t*_	1996 −0.007 (0.002)	2000 −0.029 (0.013)	2010 0.4154 (0.1870)	2010 0.3011 (0.1392)	2010 0.1778 (0.0827)
	1997 −0.006 (0.002)	2005 0.037 (0.014)			
	1998 −0.005 (0.002)				
	2011 −0.009 (0.004)				

^a^Only those coefficients where the 95 % credible interval did not contain the zero (statistically significant); ^b^mean (standard deviation)

Most of the random-effects associated to the lagged dependent variable on the region level were statistically significant. Results can be obtained from authors on request

Source: own construction

For the life expectancy model, the explanatory variables which had a (statistically) significant effect were the Gini index, −0.1836 %, the employment in high-tech, 0.001 %, the external balance, 0.0237 %, public expenditure, 0.0081 % and the expansion of 2004, 0.0051 %. As for total mortality, the significant explanatory variables with a significant effect were the GDP rate, 0.0041 %, the GDP rate (lag 1), −0.0044 %, the Gini index (lag 1), 1.0406 %, public expenditure, −0.2011 % and the crisis effect (from 2008), 0.0286 %.

For mortality due to ischemic heart disease, the significant explanatory variables which had an effect were employment in high-tech, 0.0038 %, the proportion of university students, 0.1488 %, young male (0.1876 %) and female (−0.2707 %) unemployment and public expenditure, −0.3496 %. As for standardised cancer rates, the explanatory variables with a significant effect were the Gini index (lag 1), 2.2466 %, the proportion of university students, 0.1074 % and young female unemployment, −0.1870 %. Finally, for lung cancer mortality, the explanatory variables, which had an effect on the convergence were the GDP rate (lag 2), 0.0109 %, the Gini index, 1.8337 %, the proportion of university students, 0.1181 % and young male (0.1075 %) and female (−0.2125 %) unemployment.

Table 3 shows the results of estimating the random-effects. Although there was average β -convergence for the regions of the EU-27 in the five health variables considered (i.e., the coefficient of interest, β, was negative and statistically significant) - there were discontinuities in convergence between countries, region and over time. While there was no divergence in any country, the rate of convergence in life expectancy at birth was less than the average in Estonia, Portugal, Poland and Hungary and higher in Greece, Sweden, Spain and Italy. As regards to total mortality, note that in Ireland the convergence rate was less than the average, while in Poland it was higher. With regard to mortality from ischemic heart disease, in France and the Netherlands the rate of convergence was lower than the average and in the Czech Republic it was above the average. For cancer mortality, the Czech Republic, Hungary and Bulgaria all had a convergence rate above the average. Finally, with regard to mortality from lung cancer, Germany had a convergence rate below the average, while Czech Republic and Hungary were above.

Results in relation to random-effects associated to time suggest that β -convergence did not occur in all countries with the same intensity in every moment of time. In this sense, for example, in 2010 a divergence occurs in cause-specific mortality.

Summing up, our results indicate that there was (statistically) significant β-convergence in life expectancy, total mortality and mortality (ischemic heart disease, lung cancer and cancer) among the EU-27 regions for the study period. This means that, in terms of health, there was a catching-up process between the EU-27 regions between 1995 and 2011. However, although we find β-convergence on average, we also identify significant differences in the catching-up process across both time and regions.

**Fig. 1:**
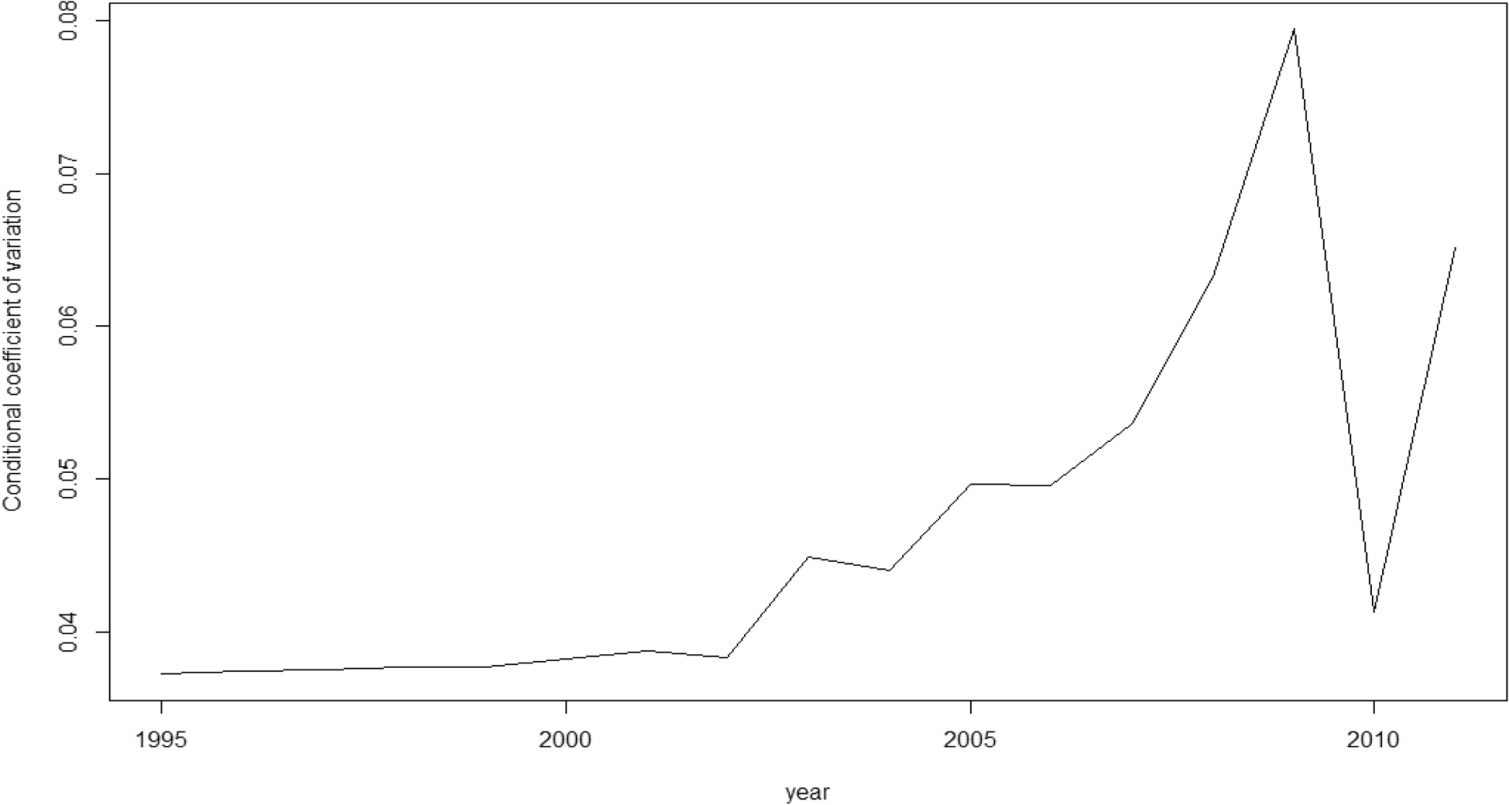
σ-convergence (Life expectancy at birth). Source: own construction

**Fig. 2:**
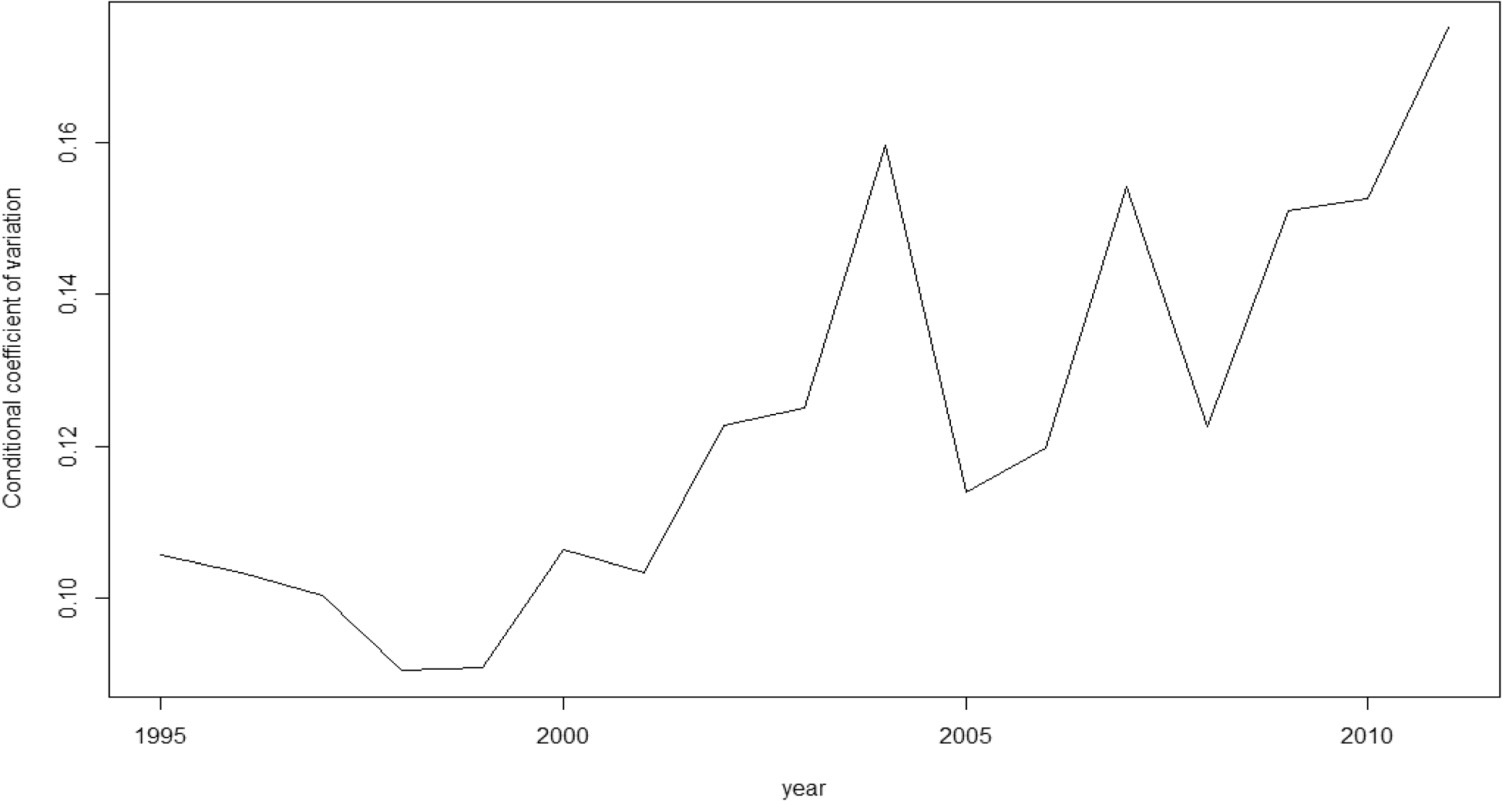
σ-convergence (Total Mortality). Source: own construction

**Fig. 3:**
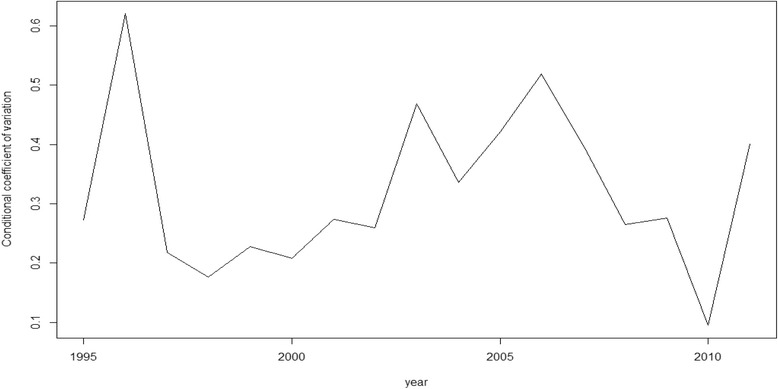
σ-convergence (Ischemic heart disease). Source: own construction

**Fig. 4:**
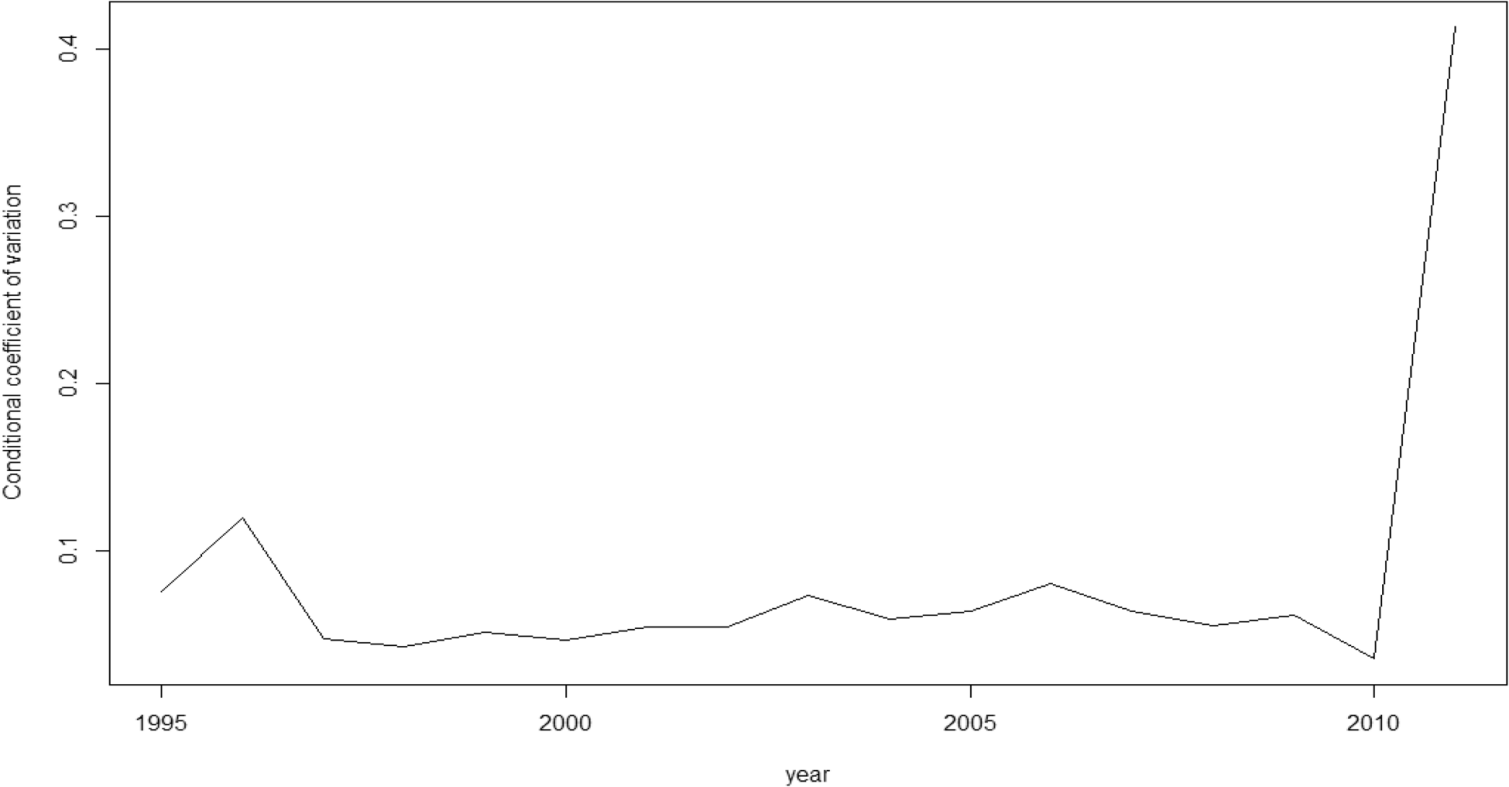
σ-convergence (Cancer Mortality). Source: own construction

**Fig. 5:**
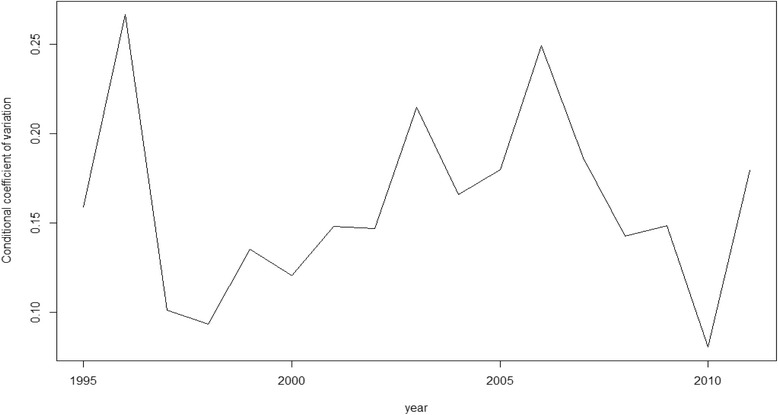
σ-convergence (Lung Cancer Mortality). Source: own construction

Nevertheless, as we said above, rather than β-convergence our objective here is, in fact, σ-convergence (Figs. 1, 2, 3, 4 and 5). The figures show the evolution on disparities in these health outcomes. From these graphs we extract the main result of this paper. First of all, note that σ-convergence did not occur in all cases (when the coefficient of variation increases). One common fact, in all figures, is that there is a sharp increase in 2010. We can relate this year with the implementation of austerity measures in the EU countries. So, even though the crisis started in 2008, we observe an impact two years later, in 2010.

Analysing the figures in more detail, we can observe a gradual increase of disparities in life expectancy from 2002 to 2009, followed by a severe decrease until to 2010, only to rise again from this year on. Total mortality is the only health output where we cannot clearly observe the sharp increase in 2010. However, from 2000, there has been a gradual increase of disparities (with some ups and downs). Mortality due to ischemic heart disease and lung cancer mortality behave similarly. Until 2006 disparities increased for both health outcomes. However, from 2006 to 2010, disparities dropped only to increase sharply again in 2010. In the case of cancer mortality, disparities had not moved a lot during the years previous to 2010, but from this year onwards, they rose sharply.

## Discussion

The main objective of this paper was to show the impact of the economic crisis on health inequalities. This impact has been analysed through the σ-convergence hypothesis (i.e. the reduction or equalising of disparities) among the EU-27 in the period 1995–2011. This aim is achieved through specifying a dynamic panel model with random-effects (time, regions and countries).

The results of our analysis show that, in terms of health, there has been a catching-up process among the EU regions. The coefficient of interest, β, was negative and significant for the five models. However, as found in a recent paper [21], we find no reduction, on average, in dispersion levels as the σ-convergence showed. The Figures (Figs.1, 2, 3, 4 and 5) of this paper account for this dispersion, showing a sharp increase from 2010 onwards. Comparing these figures with our recent paper [21], we can observe a common tendency until 2009. However, including more years in the sample implied a change in the scale level of the x-axis and, in this paper, the dispersion is not shown in percentages.

The main finding of this paper is the sharp increase in disparities in 2010 for all the health outcomes (although less so in cancer mortality). This year is associated with the implementation of austerity measures in EU countries. So, despite the crisis beginning in 2008, we observe an impact in 2010 - two years later. It was in 2010 that the European governments realised that some policies needed to be implemented in order to stabilise the economy and overcome the recession. These measures were mainly focused on public spending cuts, privatization of public services and the deregulation of markets [1]. In other words, they established austerity programmes, which affected the different sectors of the economy. In particular, healthcare budgets were drastically reduced to cut spending in this area. The result of our research here is attributed to the austerity measures applied in Europe from 2010 onwards, which negatively affected health inequalities.

The existing literature shows evidence of an increase in health inequalities during crisis periods, for both previous crises [7, 8] as well as for the current crisis [9–12]. However, in this paper, even if our findings are in line with the previous studies, it also clearly demonstrates that this effect was delayed by two years. The references cited in this paragraph (except for [11]) do not capture this time effect because they work with cross-sectional datasets, while we are able to use panel data. As a result, the evidence of this two-year delay is a contribution to the above literature.

The work may have several limitations. First, we might have chosen other variables that would have explained the health dependent variables rate of growth. We considered this possibility, but data availability was one of the main limitations. Second, the consistency of the estimates is totally dependent on the fulfilment of the hypothesis of weak exogeneity. This, in turn, depends on at least one of its requirements. Once we made the spatio-temporal adjustment, the error terms should be identically and independently distributed. In this sense, we checked the absence of autocorrelation, spatial or temporal, in the standardized residuals of all models. In addition, using cross-correlation functions, we also checked the absence of (contemporary) correlation between the error terms and each of the regressors, including lagged dependent variables in particular. Third, as in any Bayesian analysis, the choice of the prior may have a considerable impact on the results. In the second stage of the hierarchy we allowed variation on the different levels for all coefficients, i.e. we allowed all the coefficients to be random-effects. Then, we tested that the variance of the effects was equal to zero, i.e. the effects were actually fixed. Only when we rejected this null hypothesis, did we maintain the coefficient as a random-effect. Furthermore, as regards to the third stage of the hierarchy, by increasing the precision (lowering the variance) we performed sensitivity analyses to assess how the prior on the hyperparameters influences the estimation. We found no significant differences.

## Conclusion

The main objective of this paper was to show the impact of the economic crisis on health inequalities. The main finding is the sharp increase in disparities in 2010, which coincides with the austerity measures implemented in the EU countries. Our main conclusion is that these austerity measures have had an impact on socioeconomic inequalities.

## Abbreviations

BPG: external balance
CPO: conditional predictive ordinates
DIC: deviance information criteria
EMPHT: high-tech employment
EU: European Union
GDP: gross domestic product
GF: gaussian field
GMRF: Gaussian Markov Random Field
INLA: integrated nested laplace approximation
PUBEXP: public expenditure rate
RANDD: R&D - Research and Development
SPDE: stochastic partial differential equations
UFY: youth female unemployment rate
UK: United Kingdom
UMY: youth male unemployment rate
UNIV: percentage of university students

## Footnotes

1 That is to say, $CV=E\left({y}_{ijt}\right)/{\left(Var\left({y}_{ijt}\right)\right)}^{{\scriptscriptstyle \frac{1}{2}}}$, both estimated in model (1). Also note that this calculation can only be done easily following a Bayesian approach, where it is easier to make inferences about functions of parameters and/or predictions, in particular when the function is non-linear, as is in our case (i.e. the dependent variables in (1-2) were non-linear functions of the health variables).

2 We have a preliminary estimation of all models allowing variation on the three levels (country/time) for all coefficients. In the specification shown, we have provided only the best final models. In particular, we use as selection criteria, the statistical significance of random effect and the Watanabe Information Criterion [35]. Results not shown can be requested from the authors.

3 Since models differ in the dependent variable to be explained, in the explanatory variables associated (statistically significant) with the dependent variable and the random effects (also statistically significant), the estimators of the coefficients for the same explanatory variable differ between models.
