# Erratum to: Economic crisis and health inequalities: evidence from the European Union

**DOI:** 10.1186/s12939-016-0466-x

**Published:** 2016-11-04

**Authors:** Laia Maynou, Marc Saez

**Affiliations:** 1 Center for Research in Health and Economics (CRES), Universitat Pompeu Fabra, C/Ramon Trias Fargas, 25-27, Mercè Rodoreda Building, 08005 Barcelona, Spain; 2 Research Group on Statistics, Econometrics and Health (GRECS), Universitat de Girona, Campus de Montilivi, 17071 Girona, Spain; 3 CIBER of Epidemiology and Public Health (CIBERESP), Madrid, Spain

## Erratum

Unfortunately, after publication of this article [1], it was noticed that part of the ‘Acknowledgements’ section was missing. The following sentence should have been added to the ‘Acknowledgements’ section:

“This study has been developed within the PhD Programme in International Relations and European Integration of the Universitat Autònoma de Barcelona (UAB, Spain). We would also like to thank the comments of Prof. Jordi Bacaria (UAB).”

The full correct Acknowledgements section can be seen here.
